# Deep Trunk Muscle Contraction Responses Across Different Postural and Task Conditions in Chronic Nonspecific Low Back Pain: A Cross-Sectional Ultrasound Study †

**DOI:** 10.3390/jcm15218353

**Published:** 2026-10-27

**Authors:** Meltem Güneş Akıncı, Emre Bezmez, Meltem Kasar Işıklı, Ceyda Suskun, Füsun Ardıç

**Affiliations:** Department of Physical Medicine and Rehabilitation, Pamukkale University Faculty of Medicine, Kınıklı, Denizli 20100, Türkiye

**Keywords:** low back pain, ultrasound, musculoskeletal

## Abstract

**Background:** Chronic nonspecific low back pain (CNLBP) is associated with altered deep trunk muscle function, but contraction responses across different postural and task conditions remain unclear. **Objective:** This study compared contraction ratios of the transversus abdominis (TrA), internal oblique (IO), and lumbar multifidus (LM) muscles between individuals with CNLBP and healthy controls across two postural and task conditions differing in surface stability and contraction task. **Methods:** Thirty-one individuals with CNLBP and 31 healthy controls were included. TrA, IO, and LM thicknesses were assessed ultrasonographically at rest and during contraction under static (supine/prone with limb elevation) and dynamic (exercise-ball sitting with unilateral foot elevation) conditions. Contraction ratios were calculated as contracted/resting thickness. **Results:** Resting muscle thicknesses were similar between groups. The IO contraction ratio was higher under the dynamic condition than the static condition in both groups (CNLBP, *p* < 0.001; control, *p* = 0.030); no such differences were observed for TrA or LM. The static TrA contraction ratio was nominally lower in the CNLBP group [1.10 (0.25) vs. 1.20 (0.28), *p* = 0.025] but did not reach the Bonferroni-corrected threshold (*p* < 0.0083). No other between-group differences were observed. **Conclusions:** The static TrA contraction ratio was nominally lower in individuals with CNLBP but did not reach the Bonferroni-corrected threshold; the IO contraction ratio was higher under the dynamic condition in both groups. These exploratory findings suggest muscle-specific contraction responses to differing postural and task demands, and may inform future confirmatory studies on trunk stabilization in CNLBP. Simultaneous variation in body position, task demands, and support-surface stability limits causal inference regarding the independent effect of surface instability.

## 1. Introduction

Low back pain (LBP) is one of the most common musculoskeletal disorders and is associated with reduced quality of life and physical limitations [1]. It affected 619 million people worldwide in 2020 and is projected to affect 843 million by 2050 [2]. In approximately 85% of patients, no specific pathology is identified, and symptoms persisting for more than three months are classified as chronic nonspecific low back pain (CNLBP) [3]. Altered motor control and functional impairments of the trunk muscles involved in spinal stability are thought to contribute to the development and recurrence of CNLBP [4,5].

The transversus abdominis (TrA), internal oblique (IO), and lumbar multifidus (LM) muscles contribute to lumbopelvic stability and segmental control [6,7]. While the TrA and IO contribute to trunk stabilization as deep abdominal muscles, the LM is particularly involved in the control of intersegmental movement [6,8]. Reduced muscle thickness and altered contraction patterns have been reported in these muscles in individuals with CNLBP [5,9]. Accordingly, spinal stabilization exercises are widely used in the management of chronic LBP, and unstable surfaces such as exercise balls are increasingly incorporated to increase trunk muscle demand. Exercise-ball sitting has been shown to increase TrA and IO muscle thickness compared with chair sitting in both healthy individuals and those with chronic LBP [10,11]. Although resting TrA thickness may be similar between groups, the increase during unstable sitting may be smaller in individuals with chronic LBP [12]. Similarly, exercise-ball sitting has been reported to increase LM cross-sectional area in both individuals with and without LBP [13].

Musculoskeletal ultrasonography is increasingly used because it is inexpensive, radiation-free, and provides information on muscle thickness, cross-sectional area, and function [14]. The contraction ratio (CR), defined as contracted-to-resting muscle thickness, has been recommended for assessing muscle function [15]. Previous studies have shown that changes in muscle thickness during low-level contractions are associated with electromyographic activity and are therefore considered to reflect muscle activation [16,17]. Ultrasonography allows muscle assessment at rest and during contraction under static and dynamic conditions. TrA, IO, and LM CR assessment during exercise-ball tasks has been shown to be valid and reliable in individuals with LBP [18,19].

Therefore, this study aimed to compare the contraction ratios of the TrA, IO, and LM muscles between individuals with CNLBP and healthy controls across two distinct postural and task conditions: a supine/prone static condition involving limb elevation tasks, and an exercise-ball sitting dynamic condition involving unilateral foot elevation. Understanding muscle-specific responses across these conditions may help inform the selection of exercise conditions in the rehabilitation of individuals with CNLBP.

## 2. Methods

### 2.1. Study Design

This cross-sectional study was conducted at the Physical Medicine and Rehabilitation outpatient clinic of Pamukkale University between May 2024 and May 2025. Ethical approval was obtained from the Pamukkale University Non-Interventional Clinical Research Ethics Committee before the initiation of the study (02.05.2024/E-60116787-020-521162). The study was conducted in accordance with the Declaration of Helsinki and reported in accordance with the Strengthening the Reporting of Observational Studies in Epidemiology (STROBE) guidelines.

### 2.2. Participants

An a priori sample size calculation was performed using G*Power version 3.1.9.7. The effect size was derived from the between-group difference in transversus abdominis muscle thickness change reported by Arab et al. in individuals with chronic low back pain and healthy controls [12]. Based on a Cohen’s d of 0.89, a two-tailed independent-samples t-test, an alpha level of 0.05, 90% statistical power, and a 1:1 allocation ratio, a minimum sample size of 56 participants (28 per group) was required. To account for potential incomplete or missing data, the target sample size was increased by 10% to 62 participants (31 per group). Although this effect size was derived from a study reporting muscle thickness change rather than contraction ratios, no prior studies reporting effect sizes specifically for between-group differences in TrA contraction ratios in individuals with CNLBP were available at the time of the study design. Given that the contraction ratio is calculated directly from muscle thickness measurements, the effect size reported by Arab et al. was considered the most appropriate available proxy for sample size estimation. The adequacy of this approach is acknowledged as a limitation of the a priori calculation.

The inclusion and exclusion criteria for the study groups were defined as follows. The CNLBP group included individuals aged 20–70 years with LBP localized between the 12th rib and the gluteal folds for at least three months. Individuals with nerve root compression, radicular pain, a history of spinal surgery, neurological deficits, rheumatic or neuromuscular diseases, or a history of lower extremity injury were excluded. The control group included individuals aged 20–70 years with no history of low back, back, or lower extremity pain within the previous six months; individuals with spinal region pain within the previous six months or neuromuscular disease were excluded. Written informed consent was obtained from all participants.

### 2.3. Outcome Measures

In the CNLBP group, pain intensity was assessed using the Visual Analog Scale (VAS) [20], and disability was evaluated using the Oswestry Disability Index (ODI) [21]. Physical activity levels of all participants were assessed using the Tegner Activity Scale (TAS) [22].

The VAS, a 10 cm horizontal line on which participants indicated their current level of low back pain. A score of 0 indicated the complete absence of pain, while a score of 10 represented the most severe, unbearable pain imaginable [20].

The ODI was developed to assess the degree of functional loss in individuals with low back pain. The ODI consists of 10 items covering pain intensity, personal care, lifting, walking, sitting, standing, sleeping, pain variation, traveling, and social life. Each item contains six statements, with the first statement scored as 0 and the sixth as 5. The total score is multiplied by two and expressed as a percentage, ranging from a minimum of 0 to a maximum of 100. Higher total scores indicate greater levels of disability [21].

The TAS is a scoring system ranging from 0 to 10, based on participation in daily and sporting activities. The scale ranges from 0, assigned to individuals who have discontinued activity due to injury or dysfunction, to 10, assigned to those participating in professional sports at the national team level [22].

### 2.4. Ultrasound Imaging Measurement

For each measurement condition, three measurements were obtained from the right side of each participant at the end of expiration, and the mean value was used for analysis [19,23]. Resting and contracted thicknesses of the TrA, IO, and LM were assessed under static and dynamic conditions [18,19]. All ultrasound measurements were performed by a physiatrist experienced in musculoskeletal ultrasonography, who was blinded to group allocation. Minimal probe pressure was applied throughout all measurements to avoid tissue compression. Although no formal intra-rater reliability assessment was conducted in the present study, the reliability of ultrasonographic measurements of the TrA, IO, and LM muscles during static and dynamic conditions in individuals with and without chronic low back pain has been previously established in the literature [18,19].

### 2.5. Transversus Abdominis and Internal Oblique

**Figure 1:**
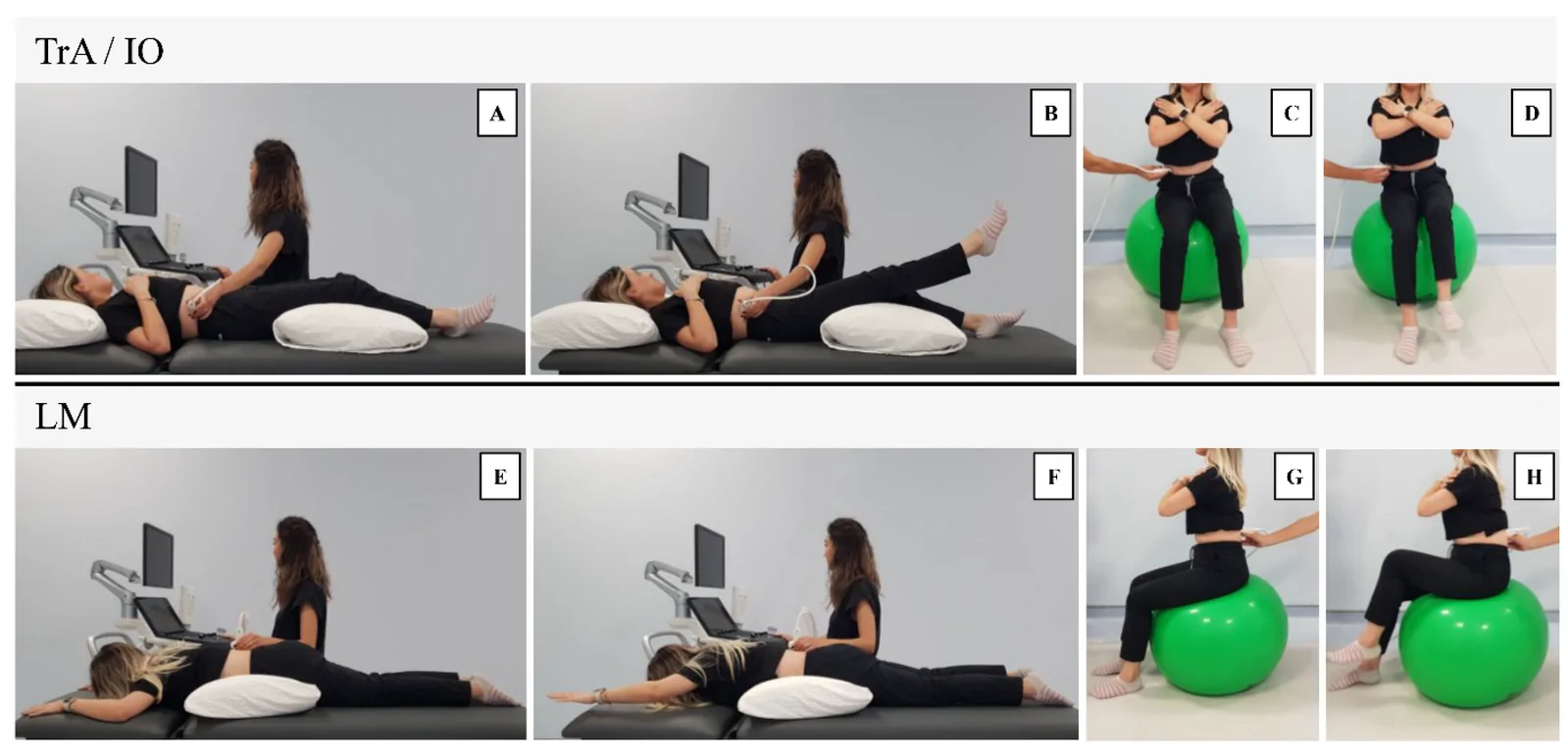
Ultrasound measurement positions for TrA/IO (**A**–**D**) and LM (**E**–**H**). (**A**,**E**): static-rest; (**B**,**F**): static-contraction; (**C**,**G**): dynamic-rest; (**D**,**H**): dynamic-contraction. Abbreviations: TrA: Transversus Abdominis, IO: Internal Oblique, LM: Lumbar Multifidus.

Muscle thickness was assessed using an 8–13 MHz linear transducer (Premier™, GE Healthcare, Chicago, IL, USA). The transducer was positioned 2 cm medial to the midpoint of the midaxillary line connecting the 12th rib and the anterior superior iliac spine [24]. Four measurement conditions were assessed: (a) Static-rest: Measurements were obtained in the supine position with a pillow under the head and another under the knees to maintain approximately 60° of hip flexion, with the arms crossed over the chest. (b) Static-contraction: While in the supine position, participants were instructed to perform an active straight leg raise by lifting the lower extremity approximately 5 cm from the examination table without flexing the knee [11,25]. (c) Dynamic-rest: Participants sat comfortably on an exercise ball (diameter: 65 cm) with the trunk upright, arms crossed over the chest, and both feet placed on the floor. (d) Dynamic-contraction: While maintaining the sitting position described in task c, participants were instructed to lift the left foot approximately 10 cm from the floor [10,18,19] (Figure 1).

### 2.6. Lumbar Multifidus

Muscle thickness was assessed using a 3–5 MHz convex transducer (Premier™, GE Healthcare, Chicago, IL, USA). The L4 and L5 spinous processes were marked with a pen. At the L4 level, after identifying the L4–L5 facet joint with the transducer, LM muscle thickness was measured [17]. Four measurement conditions were assessed: (a) Static-rest: Measurements were obtained in the prone position with a pillow placed under the abdomen to minimize lumbar lordosis. (b) Static-contraction: While maintaining the position described in task a, participants were instructed to lift the contralateral arm approximately 5 cm from the examination table. Joint angles were measured using a goniometer, with the elbow maintained at 90° and the shoulder at 120° of flexion [17,26]. (c) Dynamic-rest: Participants sat comfortably on an exercise ball (diameter: 65 cm) with the trunk upright, arms crossed over the chest, and both feet placed on the floor. (d) Dynamic-contraction: While maintaining the sitting position described in task c, participants were instructed to lift the left foot approximately 10 cm from the floor [10,18,19] (Figure 1).

**Figure 2:**
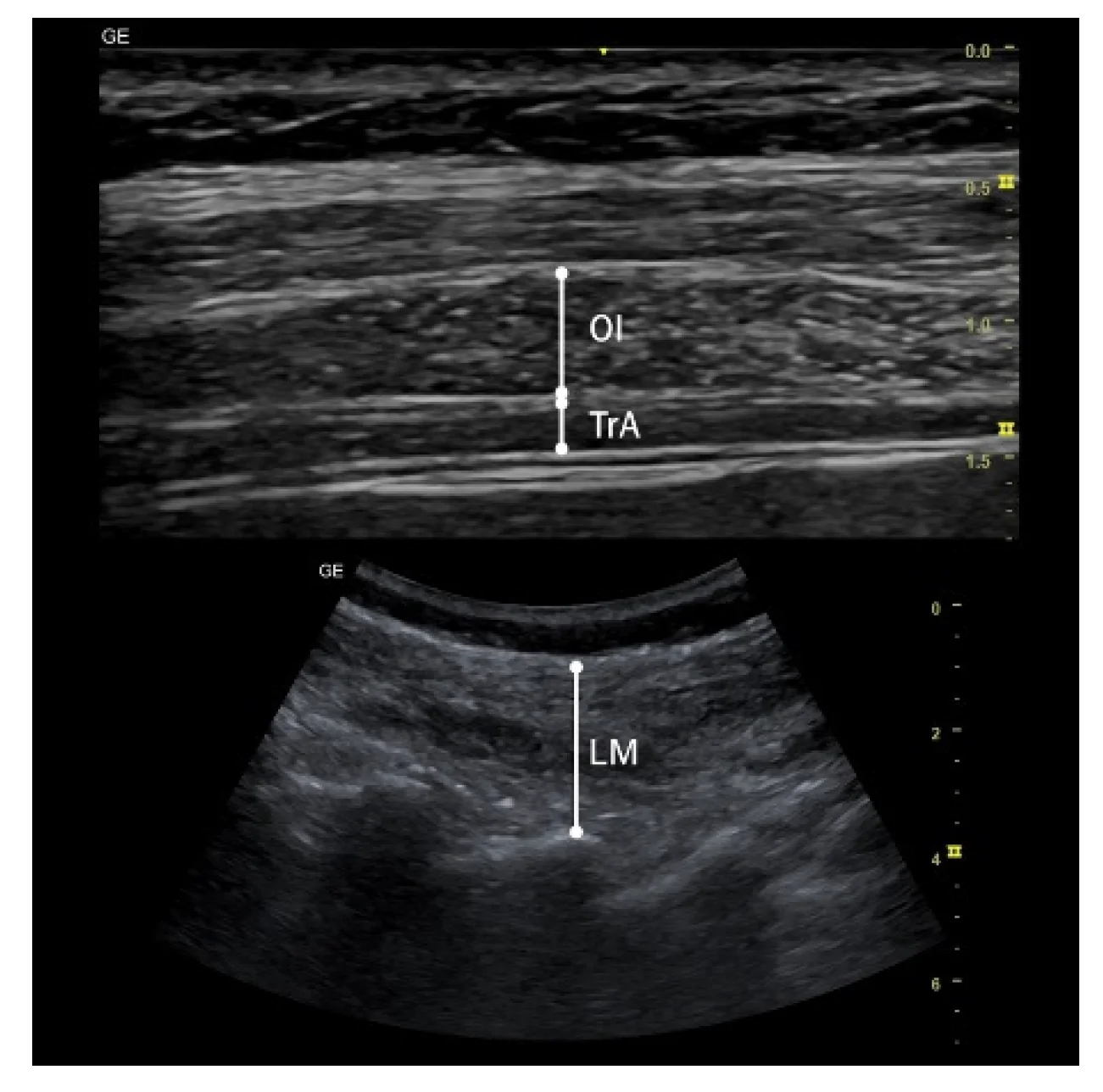
Thickness measurements of the Internal Oblique (IO), Transversus Abdominis (TrA), and Lumbar Multifidus (LM) muscles.

TrA and IO muscle thicknesses were measured in millimeters (mm) as the distance between the superficial and deep hyperechoic fascial borders, perpendicular to the muscle fibers [24]. LM muscle thickness was measured as the distance from the level of the facet joint to the interface between the muscle and subcutaneous tissue [17]. The CR for each muscle was calculated by dividing muscle thickness during contraction by resting muscle thickness [19,27] (Figure 2).

### 2.7. Statistical Analysis

Statistical analyses were performed using IBM SPSS Statistics, version 25 (IBM Corp., Armonk, NY, USA). Normality of continuous variables was assessed using the Shapiro–Wilk test. Continuous data were presented as mean ± standard deviation (SD) or median (interquartile range [IQR]), as appropriate. Between-group comparisons were performed using the independent-samples t-test for normally distributed variables and the Mann–Whitney U test for non-normally distributed variables. Categorical variables were compared using the chi-square test. Within-group comparisons of static and dynamic CRs were performed using the Wilcoxon signed-rank test. Effect sizes were calculated as r = |Z|/√N for Mann–Whitney U tests and Hedges’ g for the independent-samples t-test. A *p* value < 0.05 was considered statistically significant for primary analyses. To account for multiple between-group comparisons of contraction ratios (six comparisons across three muscles and two conditions), Bonferroni correction was applied, yielding an adjusted significance threshold of *p* < 0.0083. When multiple comparisons are performed simultaneously, the probability of obtaining at least one false-positive result increases; Bonferroni correction was therefore applied to control the family-wise error rate. Given the exploratory nature of this study, uncorrected *p* values are reported alongside the Bonferroni-corrected threshold to allow transparent interpretation of all findings.

## 3. Results

Demographic and clinical characteristics and resting muscle thicknesses are presented in Table 1. No significant between-group differences were observed in age, gender, body mass index (BMI), activity level, or resting muscle thicknesses (all *p* > 0.05).

Within-group comparisons of static and dynamic CRs are shown in Table 2. In both groups, the IO CR was significantly higher under the dynamic condition than under the static condition (CNLBP, *p* < 0.001; control, *p* = 0.030). No significant differences between static and dynamic conditions were observed for the TrA or LM (*p* > 0.05).

**Figure 3:**
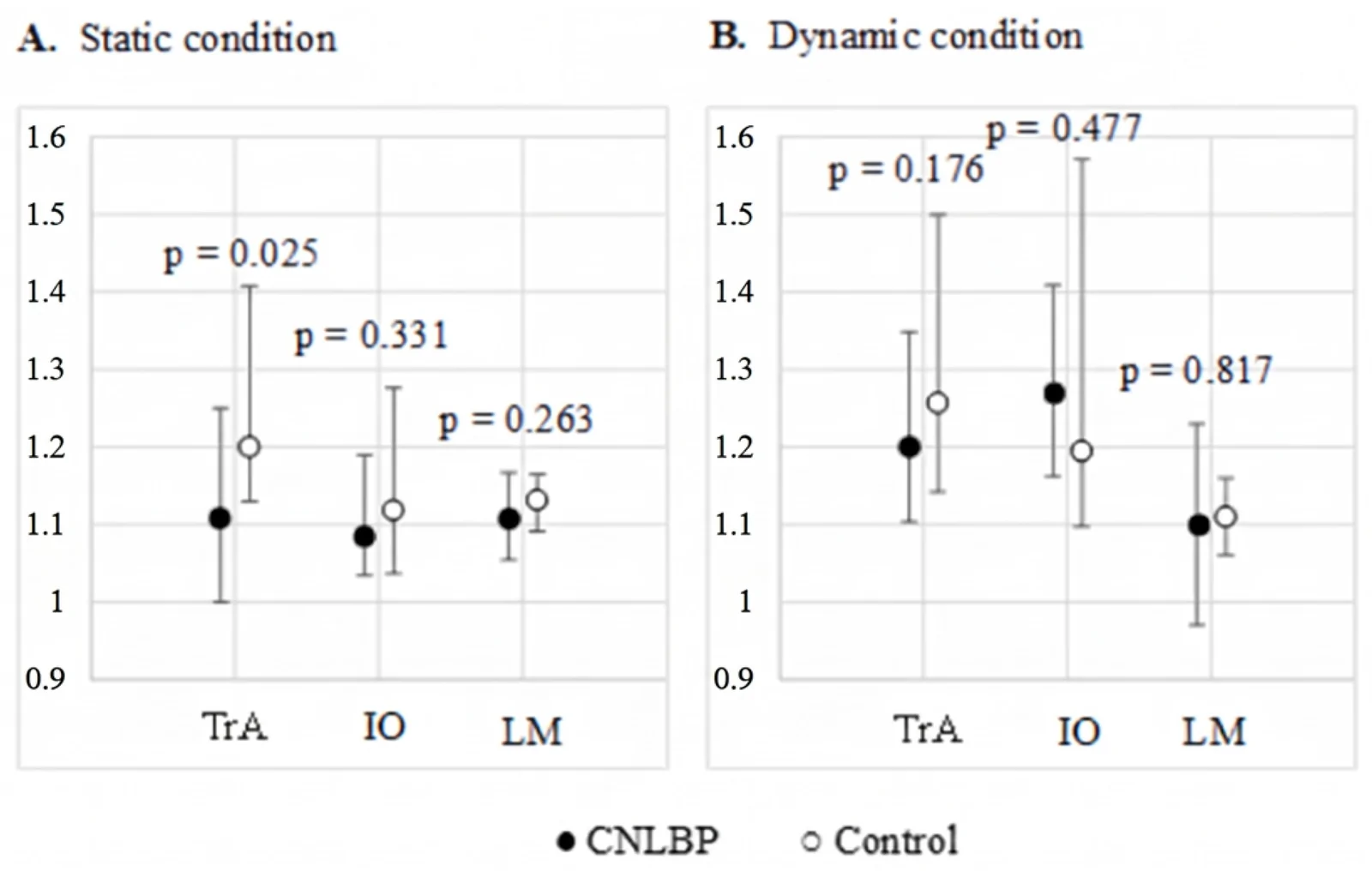
Comparison of contraction ratios between the CNLBP and control groups during static and dynamic positions.

Between-group comparisons of CRs are presented in Figure 3. The static TrA CR was nominally lower in the CNLBP group than in the control group [1.10 (0.25) vs. 1.20 (0.28); U = 321.0, *p* = 0.025, r = 0.29]; however, this difference did not reach the Bonferroni-corrected significance threshold of *p* < 0.0083 and should therefore be interpreted as an exploratory finding. No significant between-group differences were observed for static IO (U = 411.5, *p* = 0.331, r = 0.12), static LM (U = 401.0, *p* = 0.263, r = 0.14), dynamic TrA (U = 384.5, *p* = 0.176, r = 0.17), or dynamic IO (U = 430.0, *p* = 0.477, r = 0.09). Dynamic LM CR was also comparable between groups (1.108 ± 0.136 vs. 1.115 ± 0.051; Welch’s t(38.14) = −0.233, *p* = 0.817, Hedges’ g = −0.06).

Median (IQR) values were compared with the Mann–Whitney U test; dynamic LM is shown as mean ± SD and compared with an independent-samples t-test. Abbreviations: CNLBP: Chronic nonspecific low back pain, TrA: Transversus Abdominis, IO: Internal Oblique, LM: Lumbar Multifidus.

## 4. Discussion

In this study, the contraction responses of the TrA, IO, and LM muscles under static and dynamic conditions were compared between individuals with CNLBP and healthy controls. In both groups, the IO CR was significantly higher under the dynamic condition than under the static condition, whereas no significant differences between conditions were observed for the TrA or LM. Between-group comparisons showed that the static TrA CR was nominally lower in individuals with CNLBP, although this difference did not reach the Bonferroni-corrected significance threshold and should be considered an exploratory observation. No significant between-group differences were observed in the CRs of the TrA, IO, or LM under dynamic conditions. In addition, the similar baseline resting muscle thicknesses between the groups suggest that functional responses during specific tasks may be more affected than the structural characteristics of these muscles in CNLBP.

The IO is one of the deep abdominal muscles that contributes to trunk stabilization, and its task-dependent response may be influenced by increased postural demand [10,11]. Therefore, evaluating the IO response under static and dynamic conditions may help clarify its role in lumbopelvic control. Arab et al. demonstrated that IO muscle thickness assessed by ultrasonography increased significantly more during sitting on an exercise ball than during sitting on a chair in both individuals with chronic LBP and healthy controls [12]. In another study including only women, IO muscle thickness increased significantly in the chronic LBP group when transitioning from the supine position to sitting on an exercise ball, whereas in the control group, a significant increase was observed when transitioning from sitting on an exercise ball to a more unstable position involving single-leg elevation [28]. While previous studies have primarily evaluated changes in IO muscle thickness across different postures, the present study assessed muscle response relative to rest using the CR. In contrast, Kalantari et al. found no significant difference in IO activity between static and dynamic standing postural tasks in older adults and attributed this finding to age-related changes in muscle morphology, sarcopenia, and motor control [29]. In the present study, the higher IO CR during the dynamic condition than during the static condition in both groups is consistent with posture-related increases in muscle thickness reported previously. However, because the two conditions in the present study differed simultaneously in both surface stability and contraction task, it is not possible to determine whether this difference reflects a response to surface instability per se, to the different contraction task, or to a combination of both factors. Future studies using a controlled design that isolates surface instability from contraction task will be needed to clarify the independent contribution of each.

The TrA, another deep abdominal muscle, has been reported to play an important role in enhancing lumbar stability [16,30]. In a study by Arab et al. investigating the use of an unstable surface to increase TrA activity, TrA thickness increased during sitting on an exercise ball compared with sitting on a chair in both individuals with chronic LBP and healthy controls. However, the between-group comparison showed that the increase in TrA thickness when transitioning to sitting on an exercise ball was significantly smaller in the LBP group than in the healthy group [12]. Morelhão et al., in a study including only women, found no between-group difference in TrA muscle thickness in the supine position or while sitting on an exercise ball. However, in the more unstable position involving lifting the left lower extremity approximately 10 cm from the floor while sitting on the exercise ball, TrA thickness was significantly lower in the LBP group [28]. The findings of both studies suggest that, in chronic LBP, the adaptive response of the TrA may be particularly affected as postural stabilization demands increase. Similarly, Kalantari et al. reported that TrA activity increased during the dynamic task only in the healthy control group, whereas no significant change was observed between static and dynamic standing tasks in the LBP group. However, no overall between-group difference in TrA activity was detected [29]. In the present study, no significant difference in TrA CR was observed between static and dynamic conditions in either group. Between-group comparisons showed a nominally lower TrA CR in individuals with CNLBP only under the static condition (*p* = 0.025), although this did not survive Bonferroni correction for multiple comparisons (threshold *p* < 0.0083) and should be regarded as hypothesis-generating rather than confirmatory evidence. No significant between-group difference was observed under the dynamic condition. The discrepancy between the present findings and those of previous studies may be related to differences in assessment methods. Previous studies primarily evaluated posture-related changes in TrA muscle thickness, whereas the present study used the CR. In addition, activation of the TrA by the unstable surface during the resting phase of the dynamic condition may have limited the additional increase in muscle thickness elicited by leg elevation and, consequently, reduced the between-group difference in CR.

Alterations in LM muscle function have been reported in individuals with LBP [31], and LM dysfunction has been suggested to contribute to the recurrence of LBP [17,32]. Scott et al. demonstrated that sitting on an exercise ball increased LM activity compared with sitting on a stable surface in both individuals with chronic LBP and healthy controls, although no significant between-group difference was observed [13]. Similarly, Fortin et al. reported that the LM contraction response during submaximal contraction was comparable between individuals with and without LBP, while the contraction-induced increase in muscle thickness was greater in the prone position than in standing [33]. In the present study, no significant differences in LM CR were observed either between static and dynamic conditions or between individuals with CNLBP and healthy controls. This finding may be explained by the possibility that the LM was already activated for postural stabilization during unstable sitting, thereby limiting the relative increase in muscle thickness induced by additional contraction.

This study has several limitations. First, its cross-sectional design does not allow causal relationships to be established between CNLBP and differences in muscle contraction responses. Second, muscle function was assessed using ultrasonographic muscle thickness and CR without concurrent EMG assessment. In addition, because the assessments were limited to specific static and dynamic tasks, the findings may not be directly generalizable to functional activities involving greater postural demands or those that more closely reflect activities of daily living. Finally, the relatively limited sample size may have reduced the ability to detect smaller between-group differences. Future studies should include larger samples and investigate TrA, IO, and LM muscle responses using bilateral ultrasonographic assessments combined with appropriate EMG methods across different levels of stability and during more functional tasks. Although Bonferroni correction was applied to the six between-group contraction ratio comparisons, the sole nominally significant finding (static TrA CR, *p* = 0.025) did not reach the corrected threshold of *p* < 0.0083. Accordingly, this finding should be interpreted cautiously and regarded as exploratory, warranting replication in larger, adequately powered confirmatory studies. A methodological limitation inherent to the study design also warrants explicit acknowledgement: the static and dynamic conditions differed simultaneously in both body posture and contraction task, which prevents isolation of the independent effect of surface instability on muscle contraction responses. The observed between-condition differences in IO CR may therefore reflect the influence of postural change, task change, or their interaction, rather than surface instability alone. Future studies employing designs that decouple these variables—for example, by assessing the same contraction task across surfaces of varying stability, or the same posture with systematically varied stability levels—would allow more definitive conclusions regarding the specific contribution of surface instability to deep trunk muscle contraction responses.

## 5. Conclusions

In this study, the contraction responses of the TrA, IO, and LM muscles under static and dynamic conditions were compared between individuals with CNLBP and healthy controls. The IO CR increased under the dynamic condition compared with the static condition in both groups, whereas no significant changes were observed in the TrA or LM between conditions. Between-group comparisons showed a nominally lower TrA CR under the static condition in individuals with CNLBP; however, this difference did not reach the Bonferroni-corrected significance threshold and should be interpreted as an exploratory finding. No statistically significant between-group differences were observed in TrA, IO, or LM CRs under the dynamic condition; however, the absence of significant differences should not be interpreted as evidence of equivalence between groups. The similarity in baseline resting muscle thicknesses further suggests that, in CNLBP, functional muscle responses during specific tasks may be more affected than structural muscle characteristics. These exploratory results may inform hypothesis generation for future confirmatory studies examining trunk muscle contraction responses across different stability conditions. Future studies should employ more comparable tasks and incorporate concurrent electromyographic (EMG) assessment to better characterize muscle activation patterns. Ultrasonography may provide a non-invasive and clinically applicable method for such assessments. However, since body position, task demands, and support-surface stability were altered simultaneously across conditions, the observed differences cannot be attributed solely to surface instability; future studies employing more comparable tasks are needed to clarify the independent contribution of each factor.

## Figures and Tables

**Table 1:** Comparison of baseline demographic and clinical characteristics and resting muscle thicknesses.

Variable	CNLBP (*n* = 31)	Control (*n* = 31)	Test Statistics	*p*-Value
Age (years), median (IQR)	51 (15)	43 (17)	U = 409.5	0.316 ^a^
Gender, *n* (%) Female Male	25 (80.6%) 6 (19.4%)	20 (64.5%) 11 (35.5%)	χ^2^ = 2.026	0.155 ^b^
BMI (kg/m^2^), median (IQR)	26.72 (7.15)	26 (5.84)	U = 430.0	0.477 ^a^
Tegner activity scale, median (IQR)	3 (1)	3 (1)	U = 470.0	0.867 ^a^
Symptom duration (months), median (IQR)	24 (12)	-	-	-
VAS, median (IQR)	6 (1)	-	-	-
ODI, mean ± SD	22.38 ± 8.81	-	-	-
TrA (mm), median (IQR)	2.80 (1.20)	2.80 (1.30)	U = 432.5	0.499 ^a^
IO (mm), median (IQR)	6.30 (3.10)	6.90 (4.10)	U = 395.0	0.228 ^a^
LM (mm), median (IQR)	28.90 (7.08)	29.60 (4.37)	U = 436.0	0.531 ^a^

^a^ Mann–Whitney U test, ^b^ Chi-square test. Abbreviations: CNLBP: Chronic nonspecific low back pain, BMI: Body mass index, VAS: Visual Analog Scale, ODI: Oswestry Disability Index, TrA: Transversus Abdominis, IO: Internal Oblique, LM: Lumbar Multifidus, IQR: Interquartile Range, SD: Standard Deviation.

**Table 2:** Within-group comparison of contraction ratios during static and dynamic conditions.

Muscle		CNLBP (*n* = 31)			Control (*n* = 31)			
	Static	Dynamic	Z	*p*-Value	Static	Dynamic	Z	*p*-Value
TrA, median (IQR)	1.10 (0.25)	1.2 (0.24)	−1.529	0.126	1.2 (0.28)	1.25 (0.36)	−0.956	0.339
IO, median (IQR)	1.08 (0.16)	1.26 (0.25)	−3.586	**<0.001**	1.1 (0.24)	1.19 (0.47)	−2.175	**0.030**
LM, median (IQR)	1.10 (0.11)	1.06 (0.17)	−0.999	0.318	1.13 (0.07)	1.12 (0.08)	−1.705	0.088

Wilcoxon signed-rank test. Abbreviations: CNLBP: Chronic nonspecific low back pain, TrA: Transversus Abdominis, IO: Internal Oblique, LM: Lumbar Multifidus, IQR: Interquartile Range. Bold values indicate statistically significant results (*p* < 0.05).

## Data Availability

The data are available from the corresponding author upon reasonable request.
